# A Customized Radiation Carrier for Recurrent Maxillary Cancer: A Clinical Report

**DOI:** 10.7759/cureus.106862

**Published:** 2026-04-11

**Authors:** Xuewei Han, Mariko Hattori, PL Ranganayakidevi S Palaniappan, Mihoko Haraguchi, Yuka Sumita, Noriyuki Wakabayashi

**Affiliations:** 1 Prosthodontics, Private Practice, Shanghai, CHN; 2 Department of Advanced Prosthodontics, Institute of Science Tokyo, Tokyo, JPN; 3 Department of Prosthodontics, SEGi University, Kuala Lumpur, MYS; 4 Department of Prosthodontics, University of Malaya, Kuala Lumpur, MYS; 5 Department of Partial and Complete Denture, The Nippon Dental University, School of Life Dentistry at Tokyo, Tokyo, JPN

**Keywords:** brachytherapy, maxillary cancers, oral cancers, radiation carrier, radiotherapy prosthesis, recurrence, trismus

## Abstract

Brachytherapy is a radical treatment option for recurrent head and neck cancer and helps restore oral function and quality of life by limiting post-treatment sequelae. Use of a radiotherapy prosthesis allows delivery of the radiation dose to the desired location and minimises irradiation to normal tissue. For a satisfactory outcome, the radiotherapy prosthesis should have good adaptability and fit. However, conventional fabrication methods cannot accurately capture inaccessible anatomical structures. This clinical report describes a case in which a preloading radiation carrier was constructed by replication of a denture with a relining technique that was delivered for a patient with recurrent maxillary cancer and severe trismus.

## Introduction

Head and neck cancer is the sixth most common cancer worldwide [1]. Even with the best available therapy, 20%-40% of patients develop locoregional recurrence [2]. The choice of treatment for recurrence of this type of cancer depends on the site and disease stage, the treatment history, and the health status of the patient [2-4]. Low-dose-rate brachytherapy, which is distinguished by a dosage gradient with a locally concentrated dose, is used as a supplemental treatment for patients who have recurrent tumors, are not surgical candidates, need salvage after surgery, or require symptom control [5]. In selected patients, it may also serve as a radical treatment. However, concerns persist regarding radiotherapy-related sequelae in patients with recurrent disease, including radionecrosis, dysphagia, trismus, disease progression, and reduced quality of life [6], which is a result of reduced radiation tolerance of healthy tissue [7].

Various techniques for the creation of customised radiation carriers that can perform radiation dosimetry and direct radiation doses to the target lesion have been reported [8-13]. To our knowledge, conventional procedures require the patient to be conscious and coherent [14]. Moreover, there are many clinical reports on techniques that have been developed for loading a radiation carrier that minimise radiation time and exposure for both radiation oncologists and patients, but come at the cost of complexity and patient dissatisfaction during treatment [10,11,13,15-17]. Because few cases of preloading radiation carriers have been reported [9,18,19], it would be of interest to determine the therapeutic effect of various preloading radiation carrier cases.

This report describes a case of recurrent tumor in which the primary lesion had been surgically resected at the initial treatment, and in which a radiotherapy appliance was urgently created using the existing obturator. Written informed consent was obtained from the patient.

## Case presentation

**Figure 1:**
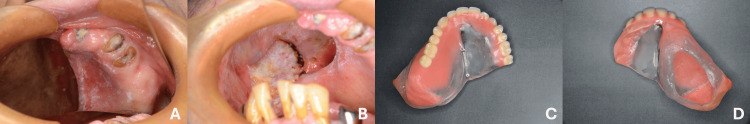
Clinical overview of maxillary defect and obturator prosthesis​ A, B) Intraoral view of the right maxillary defect in a maxillectomy patient presenting with recurrent maxillary cancer and severe trismus. C, D) Patient's existing obturator prosthesis.

A 78-year-old man was referred to the clinic for maxillofacial prosthetics in 2020 by a radiation oncologist for the fabrication of a radiotherapy appliance because of tumor recurrence in the right maxilla (Figure 1A, 1B).

He had a history of squamous cell carcinoma (T3N0M0) in the right posterior maxilla region, staged according to the tumor-node-metastasis (TNM) classification in 2002. The tumor had a diameter of 18 mm and was treated with carboplatin chemotherapy and radiotherapy (2.5 Gy / fraction 4 times weekly, total 50 Gy). Then a maxillectomy was also performed with reconstruction using a split-thickness skin graft. In 2003, the patient underwent repeated maxillectomy (Aramany class Ⅰ). The lesion was repaired by undermining the zygomatic skin and primary closure. He had been using an obturator (Figure 1C, 1D) fabricated at the clinic and regularly visited the clinic for adjustments. Tumor recurrence was detected in 2019, and external beam radiotherapy (total dose, 20 Gy) was administered at another institution. Because of radiation-induced trismus, the obturator was adjusted to a flat shape. In 2020, the tumor showed further progression.

The tumor recurrence measuring 35 × 20 × 7 mm was confirmed. Trismus was confirmed, with the mouth opening in the range of one finger. The tumor site was difficult to access due to the trismus, and the lesion had an irregular surface. The patient was in poor general condition owing to advanced age and recurrent disease.

**Figure 2:**
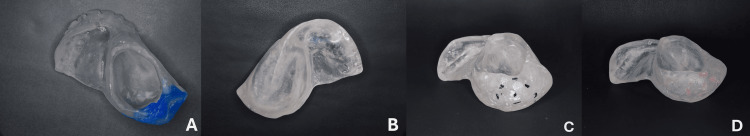
Fabrication of the brachytherapy carrier using the obturator​ A) A duplicated obturator was prepared for use as a brachytherapy carrier, and the surface facing the tumor was adjusted using soft wax. B) The adjusted surface was replaced with acrylic resin, and the occlusal surfaces of the denture teeth were trimmed flat. C) Lateral view of the carrier showing marked positions for gold-198 (^198^Au) grains (ø 0.8 mm × 2.5 mm), as designated by the radiation oncologist. D) Au grains embedded within the carrier using paraffin wax and auto-polymerising acrylic resin.

The radiation oncologist determined the location of the target site and prescribed the radiation dose. Low-dose-rate brachytherapy, consisting of a total dose of 75 Gy delivered over five days, was prescribed in 2020. No shielding was required in this case because no adjacent tissues were at risk of radiation exposure. The radiation carrier using an existing obturator (Figure 1C, 1D) was planned. The current obturator was duplicated using denture base acrylic resin (Palapress vario, Kulzer, Hanau, Germany). The fitting surface facing the tumor was relined with soft wax (Soft Plate Wax, GC Corp., Tokyo, Japan) (Figure 2A). After replacing the soft wax with acrylic resin, the teeth portion was trimmed to obtain a smooth surface (Figure 2B). The radiation oncologist then marked the position of radioactive seeds and then encapsulated seven gold-198 (^198^Au) grains (ø 0.8 mm × 2.5 mm, half-life 2.7 days) using self-polymerising resin (Unifast Ⅲ, GC Corp., Luzern, Switzerland) and wax (Paraffin wax, GC Corp., Luzern, Switzerland) within the radiation carrier (Figure 2C, 2D).

Throughout the five days of brachytherapy, which was given using the Paterson-Parker technique, the patient experienced no discomfort while wearing the carrier (day and night, except during meals). The lesion disappeared without any evidence of mucositis or ulceration in the surrounding tissues in observations over the following few days. The patient's trismus worsened in 2021 and was considered to be a side effect of radiotherapy. The patient was followed up regularly at three-month intervals for evaluation and adjustment of the obturator, which was flattened to accommodate the worsening trismus. By 2025, the patient had developed severe limitation of mouth opening; however, oral intake of soft foods remained possible.

## Discussion

Conventional fabrication of a prosthesis for radiotherapy [14] can be challenging when the patient has trismus and a lesion site that is inaccessible for impression-making, adjustment, and good adaptability, all of which are essential for successful delivery of a radioactive source and a good therapeutic outcome. In dentulous cases, the occlusal relationship helps to improve the retention and stability of a radiotherapy prosthesis [8]. In this case, an appliance with trimmed teeth functioned as an occlusal guide, facilitating insertion and removal and providing stable positioning without the interference encountered in afterloading techniques. A clear acrylic resin was used to check the adaptability of the prosthesis. A replica carrier and relining technique preserved most of the features of the patient's current obturator and had the benefits of accuracy and fixation of the radioactive source. The relining technique absorbs the pressure of the denture base, relieves pain, improves denture adaptation, and reduces trauma [20]. The soft wax used in this case is characterised by good compatibility and mucosal resilience, formation of a functional denture margin, and resistance to deformation, which augments lip support for a removable denture [21].

Prosthodontists should be mindful of several points during carrier fabrication. Because the radiotherapy prosthesis is not designed to be used when eating, the artificial teeth can be trimmed off to avoid the risk of injury to the treatment site. In addition, any difficulties with insertion or removal of the radiotherapy prosthesis due to trismus must be considered and monitored during treatment to maximise rehabilitation of oral function and quality of life.

This report has several limitations. First, it describes a single clinical case, and quantitative or standardized outcome measures were not employed. Therefore, the clinical effectiveness of this technique cannot be generalized or statistically validated. Nevertheless, the present approach enabled the fabrication of a radiotherapy prosthesis and the successful delivery of radiotherapy despite severe trismus, indicating its potential clinical feasibility. We believe that this technique may be applicable to other maxillofacial prostheses by copying existing prosthetic morphology for use during radiotherapy. Further accumulation of cases and systematic evaluation are required to clarify its broader applicability and clinical value.

## Conclusions

This clinical report describes the successful use of a preloaded radiation carrier for a patient with recurrent maxillary cancer and severe trismus. The carrier was fabricated by replicating and relining the patient's existing obturator, allowing accurate adaptation to the lesion without conventional impression-taking. Trimming the denture teeth facilitated insertion and stability, even with limited mouth opening. This approach provided a practical and comfortable alternative for delivering brachytherapy in anatomically challenging cases.
